# Supplementary material for: 5-Methoxyindole, a Chemical Homolog of Melatonin, Adversely Affects the Phytopathogenic Fungus Fusarium graminearum
Source: Int J Mol Sci. 2021 Oct 12;22(20):10991. doi: 10.3390/ijms222010991 (PMC8536143; doi:10.3390/ijms222010991)
Supplement: Supplementary file 1 [file ijms-22-10991-s001.zip › Table S1.pdf]

Table S1 DNA primers used in this study

Primer name	Sequence (5'-3')
FGSG_02881-F	TAATAACCCGGAAGGCATCC
FGSG_02881-R	CCTCCACTCACCGCTTTATT
FGSG_02974-F	GGTGGCCGAGAGGATACCT
FGSG_02974-R	CCAGGCTTAGTGGTTCCCTT
FGSG_06554-F	CCTTCAGGAGGCTATCGAGAA
FGSG_06554-R	AAACCTTGGTGGCATCGAG
FGSG_06733-F	ACTTGGCGAGGACAAGGTGA
FGSG_06733-R	GGGAATAGAGTTGACTTTCCG
FGSG_12369-F	TGACCTTGGAGAGATGGGTCT
FGSG_12369-R	TGCGATTAGACTCACTTGGT
F-Actin-F	ATCCACGTCACCACTTTCAA
F-Actin-R	TGCTTGGAGATCCACATTTG
